# Effect of *Andrographis paniculata* extract and Andrographolide on the pharmacokinetics of Aceclofenac and Celecoxib in rats

**DOI:** 10.1186/s43094-022-00450-4

**Published:** 2023-01-03

**Authors:** S. J. More, S. S. Tandulwadkar, Aishwarya R. Balap, S. Lohidasan, A. Sinnathambi, K. R. Mahadik

**Affiliations:** 1 Department of Pharmaceutical Chemistry, Poona College of Pharmacy, Bharati Vidyapeeth Deemed University (BVDU), Pune, India; 2 Department of Pharmaceutical Chemistry, PES’s Modern College of Pharmacy, Pune, India; 3 Department of Pharmacology, Poona College of Pharmacy, Bharati Vidyapeeth Deemed University (BVDU), Pune, India

**Keywords:** Celecoxib, Aceclofenac, *Andrographis Paniculata Nees*, Andrographolide, Pharmacokinetics, Herb drug Interaction

## Abstract

**Background:**

In India, for the treatment of cold, fever and inflammation, people consume herbal remedies containing *Andrographis paniculata Nees* (APE) as main ingredient, along with NSAIDs. So the purpose of this study is to investigate the effect of APE and pure andrographolide (AN) on the pharmacokinetic of with aceclofenac (ACF) and celecoxib (CXB) after oral co-administration in wistar rats. After co-administration of APE (equivalent to 20 mg/kg of AN) and AN (20 mg/kg) with ACF (5 mg/kg) and CXB (5 mg/kg) in rats, orally, drug concentrations in plasma were determined using HPLC method. Non-compartment model was used to calculate pharmacokinetic parameters like Cmax, Tmax, *t*1/2, MRT, Vd, CL, and AUC.

**Results:**

Co-administration of ACF and CXB with APE and pure AN altered the systemic exposure level of each compound in vivo. The Cmax, Tmax, MRT of CXB were increased whereas Vd and Cl of CXB were decreased significantly after co-administration of CXB with APE. Whereas co-administration of CXB with AN significantly decreased Vd, CL, and MRT of CXB. The concentration of ACF was increased significantly in co-administered groups with pure AN and APE. The AUC0-∞, AUMC0-∞, MRT, Vd and *t*1/2 of ACF were also significantly decreased in co-administered groups, hence CL of ACF was increased significantly.

**Conclusion:**

This study concludes that APE and pure AN have effect on pharmacokinetic of CXB and ACF in rat. Not only patients but medical practitioners using *Andrographis paniculata* should have awareness regarding probable herb–drug interactions with ACF and CXB.

## Background

Aceclofenac is a phenylacetic acid derivative nonsteroidal anti-inflammatory drug (NSAID). It is a potent inhibitor of cyclooxygenase and possesses anti-inflammatory and analgesic properties [1]. Celecoxib is an effective COX 2 inhibitor NSAID and act as analgesic. It is also found to be effective in the management of wide range of painful conditions [2]. *Andrographis paniculata Nees* is a herb in South Asian countries used traditionally for the treatment of cold and fever, sore throat, snake bite, and COVID 19 [3–6]. Andrographolide, a key component of *Andrographis paniculata Nees* extract (APE), has been researched on a cellular level and has been shown to have anti-inflammatory, antioxidant, and anti-cancer activities [7]. In India, people frequently consume NSAIDs and herbal treatments with APE as the primary ingredient to cure colds, fevers, and inflammation [8–11].

Several bioanalytical methods have been developed for the determination of ACF and CXB in rats and humans [12–15]. Herb–drug interaction studies of CXB with Ojeok-san, and Genistein have been reported [16, 17]. Bioanalytical methods are available for the determination of andrographolide alone and in combination with other drugs [18, 19]. Theophylline, warfarin, ibuprofen, etoricoxib, nabumetone, and naproxen interactions with APE alone and with AN have been studied before [20–24].

In this study, two new validated HPLC methods for simultaneous determination of AN with ACF and CXB in rat plasma has been developed and applied it in pharmacokinetic study in rats. The aim of the study was to explore the herb–drug interactions of APE and pure AN with ACF and CXB by studying their pharmacokinetic profiles in rats.

## Methods

### Chemicals and reagents

Celecoxib (CXB) and aceclofenac (ACF) were received as generous gifts from Cipla Pharmaceutical Pvt. Ltd. Mumbai and Jain Pharmaceutical Pvt. Ltd. Pune, Maharashtra, India. Dexketoprofen trometamol (DKT) and Mefenamic acid (MA) were obtained as generous gifts from Sun Pharmaceuticals Pvt. Ltd. Mumbai and Alkem Laboratories Pvt. Ltd. Mumbai, Maharashtra, India. Andrographolide pure (AN) was purchaased by Research Organic Pvt. Ltd, Chennai (Purity 98%). *Andrographis paniculata Nees* extract (APE) was procured by Natural Remedies Pvt. Ltd, Bangalore (Batch No. FAPEX/2013110012). All chemicals and reagents were of analytical grade and purchased from Merck Chemicals, Mumbai, Maharashtra, India. High purity deionized water was obtained from Millipore, Milli-Q (Bedford, MA, USA) water purification system.

### Animals

Male Wistar rats weighing 200–280 g were purchased from the National Institute of Biological Sciences. All animals were maintained at controlled room temperature (25 ± 2 ◦C) and humidity (60–70%) with day/night cycle (12 h/12 h). Animals were acclimated to food and water ad libitum 7 days prior to dosing. All experiments were performed as per the guidelines of CPCSEA after obtaining approval (1701/PO1C/12/CPCSEA & 1702/PO1C/12/CPCSEA) from the Institutional Animal Ethics Committee.

### In vivo pharmacokinetic study in rats

#### Drug administration and blood sampling

Animals were divided in three subgroups for both the studies as given in the Table 1. Acute toxicity studies for andrographolide were previously reported. The previous data and pilot studies were considered before finalizing the dose [25, 26]. Blood samples were collected at 0, 0.25, 0.5, 0.75, 1, 1.5, 2, 3, 4, 6, 8, 10, 12, 18, 24, 36, 48, 72 and 96 h after drug administration, for CXB and at 0.08, 0.17, 0.33, 0.50, 0.75, 1, 1.5, 2, 3, 4, 6, 8 and 10 h for ACF. Blood samples were collected by retro-orbital plexus under light ether anesthesia. The samples were transferred to EDTA tubes and centrifuged at 15,000 rpm for 20 min. Plasma was separated from blood and stored at − 80 ◦C until further analysis.

**Table 1:** Details of animal groups and dose for CXB and ACF

Group	CXB	ACF
1	CXB alone (5 mg/kg, p.o.)	ACF alone (5 mg/kg, p.o.)
2	AN with CXB (20 mg/kg + 5 mg/kg, p.o.)	AN with ACF (20 mg/kg + 5 mg/kg, p.o.)
3	APE with CXB (67 mg/kg + 5 mg/kg, p.o.)	APE with ACF (67 mg/kg + 5 mg/kg, p.o.)

*n* = 6 for each group

#### Sample preparation

To 450 μl of drug-free plasma, 10 μl each of AN (100 μg/ml) and CXB (100 μg/ml) solutions were added. To this, 50 μl of internal standard DKT (250 µg/ml) and 50 μl of 1% formic acid solution was added and vortexed for 2 min. After adding 2 ml of ethyl acetate, it was vortexed for 2 min. After centrifugation at 20,000 rpm for 20 min at 4 °C, the supernatant was separated, the solvent was evaporated and dried at 35 °C under a gentle stream of nitrogen gas. Reconstituted by adding 200 µl mobile phase and 20 µl aliquots were injected onto the HPLC system for analysis. The quality control (QC) samples were also prepared in the same manner at concentrations of 10, 200, 800 and 1000 µg/ml. Similarly, ACF samples were prepared with Diclofenac as I.S. at concentrations of 100, 450 and 900 µg/ml.

#### HPLC analysis

The HPLC analysis was performed using a Jasco PU-2080 gradient liquid chromatography instrument, with an autosampler and a UV detector UV-2075 with a Thermo Hypersil ODS column (250 × 4 mm, 5 μm). Mobile phase for CXB consisted of the mixture of solvent A acetonitrile, solvent B methanol and solvent C water, pH adjusted to 3 with ortho phosphoric acid (50: 10: 40v/v/v) for 15 min at a flow rate of 1 mL/min. The detection was performed with a UV detector at a wavelength of 254 nm. The CXB and I.S. were detected at 9.25 ± 1 and 5.40 ± 1 min respectively.

For ACF, mobile phase consisted of the mixture of solvent A acetonitrile, solvent B 0.025 M potassium dihydrogen phosphate buffer (pH 3.5) in the ratio of 55: 45(V/V), for 15 min at a flow rate of 1 mL/min. The detection was performed with a UV detector at a wavelength of 254 nm. The ACF, DCF, and MA were detected at 7.008 ± 1 min, 8.758 ± 1 min, and 11.908 ± 1 min, respectively.

#### Method validation

Method validation was executed as per the Food and Drug Administration (FDA) guidelines for quality control samples (QCs), selectivity and specificity, sensitivity, accuracy, precision, recovery, and stability of the analyte in the matrix [27]. Selectivity was checked by comparing blank plasma from six rats to the spiked plasma samples. Analysis of calibration standards was completed twice on three consecutive days. Calibration curve was generated by means of the peak area ratio of analyte to IS versus analyte concentration. A 1/*x*2 weighted linear least-squares regression model used for the calibration curve. A signal-to-noise ratio of at least 10:1 is used to calculate the limit of quantitation (LLOQ). Accuracy and precision was checked by analyzing six replicates of the QC samples at three concentrations on three successive days and relative error (RE) and relative standard deviation (RSD) were calculated. Precision limits were ≤ 20% for LLOQ and ≤ 15% for QC samples. Accuracy was found to be within ± 20% for the LLOQ and ± 15% for the QC samples. Recovery study was performed at the three levels of QC samples (*n* = 3). Matrix effects were determined by comparing the average response of the extracted analytes with the average response of the unextracted samples. The stability of CXB and ACF was carried out at three levels of QC samples (*n* = 6) in various conditions. Short-term stability was determined after storing the samples at room temperature for 2 h. Freeze–thaw stability was checked in triplicate from − 20 to 25 °C. Long term stability was performed by keeping the plasma samples at − 20 °C for 14 days.

#### Data analysis

The plasma drug concentration versus time plot was determined by a non-compartment model using WinNonlin software (Pharsight Corporation, Mountain View, CA, USA). The highest plasma concentration Cmax and Tmax were finalized directly from the independent plasma concentration–time data. The area under the curve (AUC0-t) was estimated with the linear trapezoidal rule, by extrapolating to infinity (AUC0-∞) from the last concentration detected using the elimination rate constant (ke) calculated by linear regression of the drug concentration–time curve. Apparent elimination half-life (*t*1/2) was determined from *t*1/2 = 0.693/ke, total body clearance (Cl) as dose/AUC0-∞, and apparent volume of distribution (Vd) as Cl/ke. One-way ANOVA (Bonferroni post-test) was applied to the results and significant differences were considered at *P < 0.05, **P < 0.01, ***P < 0.001.

## Results

### Method development

**Fig. 1:**
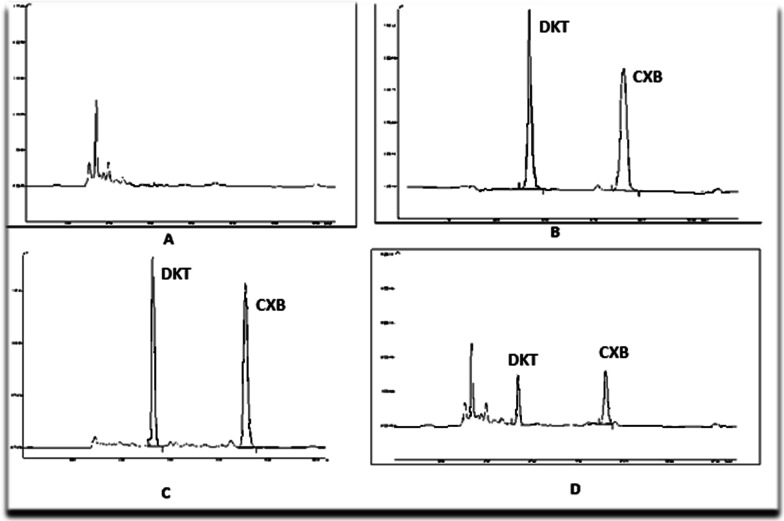
Chromatogram of **A** blank plasma sample **B** blank plasma sample with DKT (IS) **C** plasma sample spiked with I. S. DKT (10 µg/ml) and CXB (10 µg/ml) **D** a plasma sample obtained from rat at 1.5 h after oral administration of CXB

**Fig. 2:**
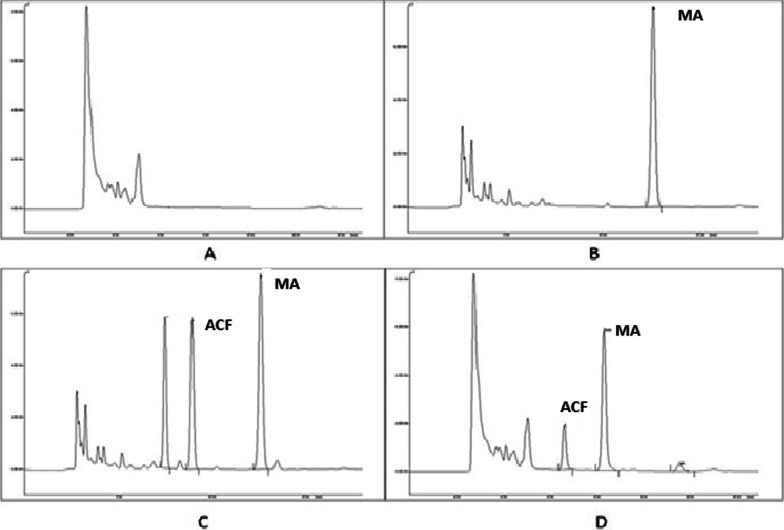
Chromatogram of **A** blank plasma sample **B** blank plasma sample with MA (IS) **C** plasma sample spiked with I. S. MA (10 µg/ml) and ACF (10 µg/ml) **D** a plasma sample obtained from rat at 1.5 h after oral administration of ACF

For CXB and ACF, the representative HPLC chromatograms of plasma samples and I.S. are shown in Figs. 1 and 2 respectively.

### Method validation

The linear response of the peak ratios versus concentrations was recorded over the plasma concentration range of 5–1000 ng/mL for CXB and 50–900 ng/mL for ACF. Calibration curve equation of the of CXB was *y* = 0.002*x* − 0.005 with correlation coefficient (*r*2 = 0.9988) and for ACF, *y* = 0.002460 ± 0.00003435 with correlation coefficient (*r*2 = 0.9987). Intra and inter batch precisions were in limit (R.S.D. ˂ 15%) and accuracy was in between 85 and 115%.

The LLOQs of CXB and ACF in rat plasma were 1 ng/mL and 50 ng/mL respectively. The intra- and inter-day precision and accuracy data for the QC samples is given in Table 2. The intra and inter-day precision for CXB was less than 2.36% and for ACF was less than 3.42, while accuracy was within ± 2.23% and ± 3.42 for CXB and ACF respectively. The matrix effect was in-between 94.12 and 97.15% for CXB and 90.25 to 94.03% for ACF at three levels of QC samples (Table 3). The matrix effect of IS was 95.97% and 91.38% for CXB and ACF respectively. The results showed that suppression or enhancement of ions by the plasma matrix was not significant under the conditions specified. The recoveries were from 1.27 to 8.90% for CXB and from 0.36 to 2.56% for ACF (Table 3). The recovery of IS of CXB at 400 ng/mL was 386.4 ± 9.37% and of ACF at 200 ng/mL was 195.5 ± 2.57%. Degradation of CXB and ACF was not significant during the 2 h, and 24 h storage at room temperature, three freeze–thaw cycles, or 14 day storage at − 70 °C. The responses varied less than 1.2% for CXB and less than 3.67% for ACF at each concentration (Table 4).

**Table 2:** Accuracy and precision for the analysis of CXB in rat plasma (*n* = 3 days, six replicates per day)

Nominal concentration ng/ml	Measured (mean ± SD)	Intra batch		Inter batch	
		% RSD	% RE	% RSD	% RE
*CXB*					
10	7.64 ± 0.55	1.58	0.60	1.25	0.04
200	187.3 ± 5.85	2.36	0.11	2.23	0.13
800	790.5 ± 6.53	1.03	0.05	1.23	0.13
1000	948.9 ± 12.04	0.97	0.08	1.04	0.12
*ACF*					
50	48.50 ± 0.48	3.424	0.077	3.424	0.089
100	95.74 ± 2.45	2.366	0.100	2.952	0.062
450	444.4 ± 4.00	0.738	0.037	0.303	0.083
900	884.8 ± 3.19	0.317	0.089	0.193	0.063

**Table 3:** Recovery and matrix effect of CXB and IS in rat plasma (*n* = 6)

Statistical variable	Nominal CXB concentration (ng/mL)				Nominal IS concentration (ng/mL)
CXB matrix effect	10	400	800	1000	400
Mean ± SD (%)	94.12 ± 2.80	96.23 ± 2.56	95.83 ± 1.90	97.15 ± 3.50	95.97 ± 2.45
RSD (%)	2.97	2.66	5.04	3.60	2.25
CXB recovery	10	400	800	1000	400
Mean ± SD (%)	8.640 ± 0.55	387.3 ± 5.85	790.5 ± 6.53	948.9 ± 12.04	386.4 ± 9.37
RSD (%)	8.90	1.51	2.71	1.27	4.57
ACF matrix effect	100	450	900		200
Mean ± SD (%)	94.03 ± 5.63	92.78 ± 3.22	90.25 ± 1.47		91.38 ± 2.63
RSD (%)	5.98	3.47	1.62		2.87
ACF recovery	100	450	900		200
Mean ± SD (%)	95.74 ± 2.45	444.4 ± 4.00	884.8 ± 3.19		195.5 ± 2.57
RSD (%)	2.56%	0.90%	0.36%		1.32%

**Table 4:** Stability studies for the determination of CXB (*n* = 5)

Spiked conc. (ng mL^−1^)	Bench top		Freeze and thaw		Long term	
	Precision % C.V	Accuracy % R.E	Precision % C.V	Accuracy % R.E	Precision % C.V	Accuracy % R.E
*CXB*						
200	0.68	0.09	0.84	0.05	1.20	0.08
1000	0.98	0.03	0.98	0.01	1.00	0.02
*ACF*						
100	2.80	0.10	3.67	0.13	1.96	0.05
900	0.17	0.03	0.24	0.04	0.13	0.03

### Pharmacokinetic herb–drug interaction study

**Fig. 3:**
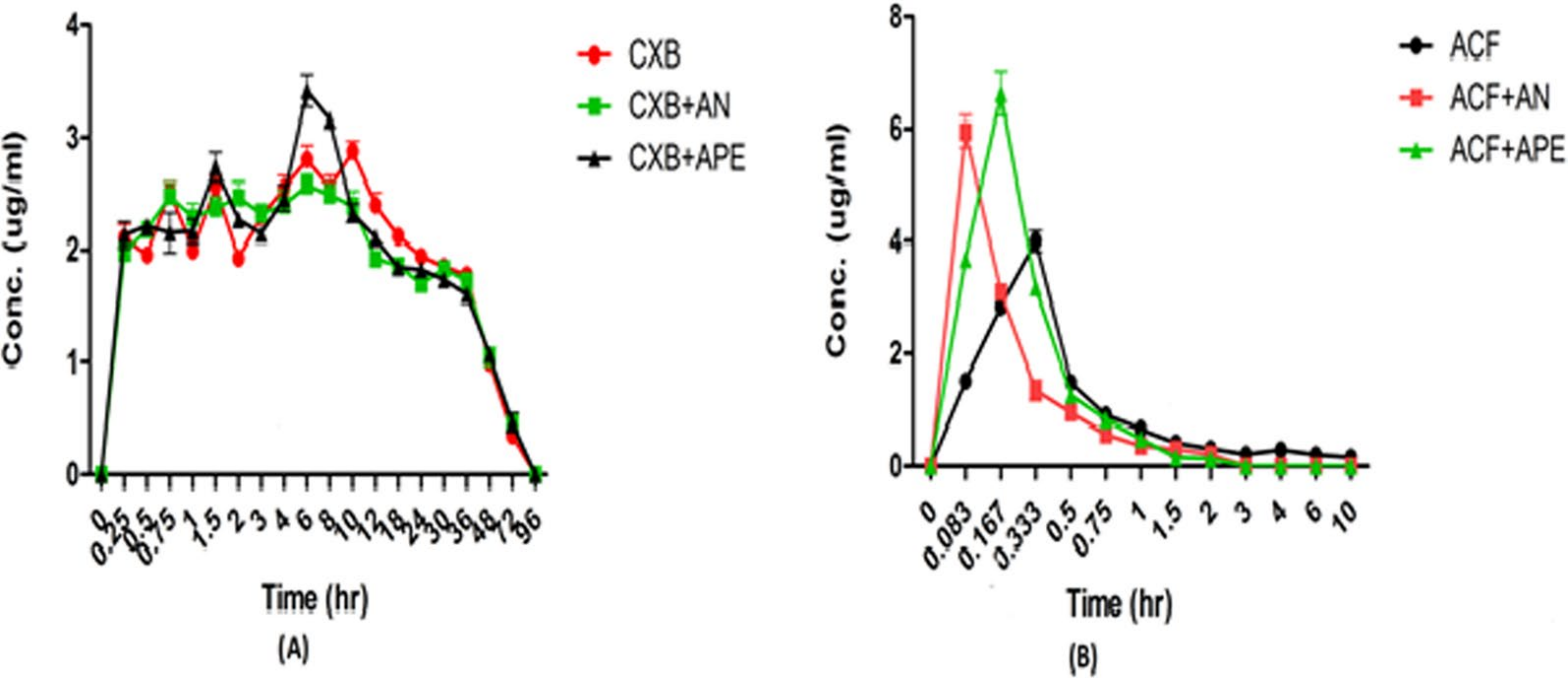
Mean plasma concentration versus time plot for CXB and ACF

For celecoxib—The plasma concentration–time plots of CXB and ACF after oral administration with or without AN and APE are shown in Fig. 3. During the studies after 24 h, significant conc. of CXB was found in the blood plasma. Hence the blood sample collection for CXB was extended up to 96 h. It increased elimination half-life of CXB which indicates plasma protein binding of CXB and should be studied further [28, 29]. The pharmacokinetic parameters of CXB after the pretreatment of rats with AN for continuous seven days reduced (*P* > 0.05) the Cmax of CXB, further when the rats were pretreated with APE at a daily dose equivalent to AN for continuous seven days resulted in a significant increase (*P* < 0.001) in Cmax of CXB. The Cmax of CXB decreased with AN pretreatment was 2.67 μg/mL and with APE pretreatment it was significantly increased to 3.40 μg/mL when compared to the control group 2.97 μg/mL. The Tmax after oral administration with AN (*P* < 0.001) was 4 h and APE was 6 h (*P* < 0.001). The elimination half-life of CXB did not show any significant effect after the pretreatment with AN and APE, but a significantly reduced to 28.89 (*P* < 0.001) and 30.57 (*P* < 0.001) in total clearance of CXB resulted following the pretreatment with APE and AN compared to control group. The mean residence time (MRT) of CXB after oral administration of AN significantly decreased to 5.97 (*P* < 0.001) and APE to 6.14 (*P* > 0.05) when compared to the control group (Table 5).

**Table 5:** Summary of pharmacokinetic data of CXB and ACF in all three groups

Parameters	CXB			ACF		
	Group A (CXB)	Group B (CXB + AND)	Group C (CXB + APE)	Group A (ACF)	Group B (ACF + AND)	Group C (ACF + APE)
C_max_ (μg mL^−1^)	2.97 ± 0.02	2.79 ± 0 .12^#^	3.40 ± 0.3**	3.86 ± 0.35	5.91 ± 0.98***	6.79 ± 0.41***
T_max_ (h)	1.5	4**	6**	0.33 ± 0.00	0.08 ± 0.00	0.17 ± 0.00
AUC _0 to t_ (μg h mL^−1^)	30.79 ± 1.66	29.47 ± 0.95	31.88 ± 0.9	4.00 ± 0.44	1.75 ± 0.08***	2.49 ± 0.13***^###^
AUC_0-∞_ (μg h mL^−1^)	145.48 ± 15.42	146.93 ± 9.84	139.33 ± 11.1	6.17 ± 0.94	2.14 ± 0.17***	2.58 ± 0.13***
AUMC _0 to t_ (μg h^2^ mL^−1^)	190.71 ± 9.51	175.94 ± 5.15	195.83 ± 4.7	11.14 ± 1.67	0.85 ± 0.03***	1.03 ± 0.05***
AUMC_0-∞_ (μg h^2^ mL^−1^)	7684.17 ± 1301.88	7173.47 ± 850.81	23,822.06 ± 785.3	71.64 ± 20.77	5.88 ± 4.02***	1.68 ± 0.04***
Vd (mL)	195.81 ± 16.95	182.52 ± 27.61	177.45 ± 28.9	832.33 ± 87.36	348.54 ± 65.62***	218.63 ± 24.01***^#^
CL (mL h^−1^)	31.60 ± 2.49	30.57 ± 4.59^#^	28.89 ± 3.1**	300.96 ± 35.53	711.79 ± 80.56***	525.85 ± 48.89***^###^
*t*1/2 (h)	31.09 ± 2.17	31.08 ± 2.67	30.49 ± 1.86	4.52 ± 0.43	1.09 ± 0.40***	0.43 ± 0.01***^##^
MRT^a^_0 to t_ (h)	6.19 ± 0.11	5.97 ± 0.03***	6.14 ± 0.04^#^	2.77 ± 0.17	0.48 ± 0.04***	0.41 ± 0.01***
MRT_0-∞_ (h)	53.02 ± 8.81	48.80 ± 4.64	56.88 ± 5.54	11.46 ± 1.73	2.66 ± 1.65***	0.65 ± 0.02***

Values are expressed as one-way ANOVA followed by Bonferronis multi comparison test *n* = 6, mean ± SD ***P < 0.0001 versus Group A, **P < 0.001, *P < 0.01, ^#^*P* > 0.05

For ACF, pretreatment of rats with AN and APE for continuous seven days resulted in a significant increase (*P* < 0.001) in Cmax of ACF. Cmax of ACF increased to 5.91 μg/ml in AN co-administered group and 6.79 μg/ml in APE co-administered group from 3.86 μg/ml in ACF alone administered group. The increase in the Cmax was found to be more with APE co-administered group compared to AN co-administered group. Tmax was significantly decreased in both AN co-administered group at 0.08 h and in APE co-administered group at 0.17 h compared to ACF alone administered group at 0.33 h. Significant decrease (*P* < 0.001) in AUC0-t, AUC0-∞, AUMC0-t and AUMC0-∞ of ACF in AN and APE co-administered groups was observed. AN and APE co-administered groups showed a significant decrease (*P* < 0.001) in Vd, MRT0-t, MRT0-∞ and *t*1/2 compared to ACF alone administered group.

## Discussion

AN is one of the active constituents of the APE. APE and AN have anti-inflammatory properties and used in cold, fever and anti-inflammatory treatment. In India, people for the treatment of cold, fever, and arthritis, commonly consume herbal formulations containing APE as the main ingredient, along with NSAID drugs such as ACF and CXB. As per literature, AN has CYP1A and CYP2B inducer activity while APE has CYP1A inhibitory activity [30, 31]. CXB is largely metabolized by CYP2C9 in humans and also by CYP1A1, CYP1A2, CYP2B1, and CYP2B2 to a lesser extent [32, 33]. As CYP2C9 is not present in rats, the possible pathway of metabolism of CXB in rats may be through CYP1A1, CYP1A2, CYP2B1 and CYP2B2. ACF has been shown to be metabolized by different mechanisms in rats and humans. In human, ACF is converted into 4’-hydroxy aceclofenac through CYP2C9 enzyme by oxidation, but only traces of DCF was detected in previous studies. Whereas in the rat plasma CYP2C9 is absent and hence 4’-hydroxy aceclofenac is not detected [34, 35]. Instead, in rats, ACF after oral administration reaches the liver via the portal vein and is rapidly hydrolyzed to DCF by hepatic esterases. From the previous studies, it is evident that several CYP450 chemical inhibitors have the potential to inhibit liver microsomal esterase enzyme activity [36]. CYP450 1A inducers also suppress the carboxylesterase [37]. The hypothesis for the study was any substance influencing the CYP1A2 enzyme is likely to affect the metabolism of ACF and CXB [20]. To decrease the probability of herb–drug interaction, studies on pharmacokinetic effects on co-administration of AN and APE with ACF and CXB would be helpful.

In ACF study, it can be assumed that significant changes in the pharmacokinetic parameters of ACF may be due to the interference in the activity of esterase enzyme through CYP1A induction and CYP2C11 inhibition activity of AN and APE which needs to be confirmed. However, the appearance of DCF in both AN and APE co-administered groups clearly confirmed the conversion of ACF to DCF due to esterase enzymes. But from the AUC0-∞ the ratio of ACF and DCF which was found to be 61.36%, 45.47%, and 49.75% for groups ACF alone, AN and APE co-administered groups respectively it is evident that AN and APE reduced the conversion of ACF to DCF, and hence this reduction may be correlated with either by partial inhibition or reduction in activity of esterase enzymes although there is no direct evidence. The reduction in Vd of ACF leads to another assumption that AN and APE might have occupied the distribution sites of ACF thus the increase in Cmax and Cl. Hence ACF is eliminated faster and it might have not been available for further conversion into DCF. These assumptions however need to be confirmed.

## Conclusion

New, simple, sensitive validated HPLC methods were developed for the quantification of CXB and ACF in rat plasma and applied in pharmacokinetic herb–drug interaction study of CXB and ACF with AN and APE. In CXB pharmacokinetic study, an increase in Cmax and Tmax of CXB was observed. A significant effect was observed on the elimination of the drug, Cl was reduced and *t*1/2, MRT was increased. ACF pharmacokinetic results showed that the concentration of ACF was increased significantly in co-administered groups with pure AN and APE. The AUC0-∞, AUMC0-∞, MRT, Vd, and *t*1/2 of ACF were also significantly decreased in co-administered groups, hence Cl of ACF was increased significantly. AN and APE decreased the AUC0-∞ and increased Vd and Cl of the DCF. The results showed that APE affected the pharmacokinetics of CXB and ACF. Administration of APE along with CXB might result in an increase in the bioavailability of CXB and can lead to toxicity. Further studies should be done to understand the mechanism, the effect of other herbal ingredients of APE on CXB and to predict the interaction in humans. The changes in pharmacokinetic parameters of ACF may be due to the interference of partial inhibition in the activity of esterase enzyme through CYP1A induction and CYP2C11 inhibition activity of AN and APE which needs to be confirmed. In addition, AN and APE might have occupied the distribution sites of ACF, thus ACF is eliminated faster and it might have not been available for further conversion into DCF.

The study concludes that AN and APE have definite interactions with the pharmacokinetics of CXB & ACF. Co-administration of APE or AN may lead to a reduction in the potency of CXB & ACF and hence pharmacodynamic study will be more helpful for further understanding. The study provides a base for the rational use of APE in the management of cold, fever and, inflammation along with CXB and ACF. Patients and medical practitioners using AP should have awareness about its herb–drug interaction with CXB and ACF.

## Data Availability

The data that support the findings of this study are available from the corresponding author, upon reasonable request.

## Abbreviations

AP: *Andrographis Paniculata Nees*
AN: Andrographolide
APE: *Andrographis Paniculata Nees* Extract
ACF: Aceclofenac
CXB: Celecoxib
NSAID: Nonsteroidal Anti-inflammatory Drug
DKT: Dexketoprofen trometamol
MA: Mefenamic acid
HPLC: High Performance Liquid Chromatography
CPCSEA: Committee for the Purpose of Control and Supervision of Experiments on Animals
*p.o**.*: Orally
EDTA: Ethylenediaminetetraacetic acid
QC: Quality control
I.S.: Internal Standard
R.S.D.: Relative standard deviation
AUC: The area under the concentration–time curve
CL: Clearance
ke: Elimination rate constant
*t*1/2: Half-life
Cmax: Maximum serum concentration
Tmax: Time to maximum concentration
MRT: Mean residence time
Vd: Volume of distribution
AUMC: Area under the first moment curve
ANOVA: Analysis of variance

## Declarations

### Plant extract

*Andrographis paniculata* extract; Source or Supplier: Natural Remedies Pvt. Ltd. Bangalore.

### Batch no

FAPEX/2013110012; All the quality control tests like identification, moisture, acid insoluble ash, bulk density, tapped bulk density heavy metals, microbiology test, residual solvent analysis, pesticide residue analysis and phytochemical analysis were within limits; Phytochemical analysis performed by HPLC with stating ≈30% w/w andrographolide in the extract.
